# One‐Year Reattendance Rates to the Emergency Department for Acute Illicit Drug Exposure in Western Australia: A Retrospective Registry Study

**DOI:** 10.1111/1742-6723.70314

**Published:** 2026-07-27

**Authors:** Courtney C. Weber, Daniel M. Fatovich, Ellie M. Kotkis, Jennifer L. Smith

**Affiliations:** ^1^ Centre for Clinical Research in Emergency Medicine Harry Perkins Institute of Medical Research Perth Western Australia Australia; ^2^ East Metropolitan Health Service Perth Western Australia Australia; ^3^ School of Population and Global Health The University of Western Australia Perth Western Australia Australia; ^4^ Emergency Medicine, Royal Perth Hospital The University of Western Australia Perth Western Australia Australia; ^5^ School of Medicine The University of Western Australia Perth Western Australia Australia

**Keywords:** emergency department, harm reduction, illicit drugs, registries, toxicology

## Abstract

**Objective:**

Determine 1‐year emergency department (ED) drug‐related reattendance rates of patients enrolled in the Emerging Drugs Network of Australia across five Western Australian hospitals.

**Methods:**

Index presentations and 1‐year reattendances were extracted. Reattendance rates were calculated and stratified by confirmed novel or traditional illicit drugs.

**Results:**

Of 1141 patients, 5.0% (95% CI: 3.9%–6.4%) reattended the ED for drug exposure within 1 year. No statistical differences between novel and traditional illicit drugs were identified.

**Conclusion:**

A conservative but discernible reattendance rate with confirmed illicit drug exposure was demonstrated. Objective data can inform hospital‐based harm reduction services and reduce future drug exposure risk.

## 1 Introduction

Recreational drug‐related emergency department (ED) visits are common, resource‐intensive (e.g., ambulance attendance, intensive care admission), and potentially preventable [1, 2]. ED reattendance rates following confirmed illicit drug and/or new psychoactive substance (NPS) exposure remain poorly characterised. Understanding reattendances by drug type (illicit or NPS) may allow for targeted harm reduction interventions and potential reductions in resource use [2]. We calculated the 1‐year severe/unusual drug‐related ED reattendance rate within the Emerging Drugs Network of Australia (EDNA) registry.

## 2 Methods

EDNA is a multi‐state ED toxicosurveillance system collating suspected severe/unusual illicit drug‐related presentations. Blood samples undergo comprehensive analysis (described previously) [3]. Data were extracted from patients aged ≥ 16 years with an index (first‐recorded) ED attendance between April 2020 and March 2024, and all 1‐year registry‐enrolled reattendances from 5 Western Australian hospitals (4 metropolitan, 1 regional).

Attendances were categorised as follows: (1) confirmed NPS exposure, (2) confirmed illicit drugs (3,4‐methylenedioxyamphetamine, 3,4‐methylenedioxymethylamphetamine, cocaine, gamma‐hydroxybutyrate, heroin [6‐O‐monoacetylmorphine], lysergic acid diethylamide, methylamphetamine) without NPS exposure, and (3) neither confirmed NPS nor illicit drugs.

### 2.1 Analysis

Data are presented as ‘*n*, %’ for categorical and ‘median (quartile 1–quartile 3)’ for continuous variables. Patients (with/without reattendances) were compared using linear/logistic univariate regression. 1‐year reattendance rates (overall, drug category) were calculated using Kaplan–Meier methods, with patients censored at 1‐year follow‐up. Only the first reattendance was analysed. Mortality information was unavailable. A *p*‐value of < 0.05 was considered statistically significant. Analyses were conducted using SAS software V9.4 (SAS Institute Inc., USA).

EDNA is registered with the Australian and New Zealand Clinical Trials Registry (ACTRN12621001234808). Ethics approval, including waiver of consent, for the registry was granted by the South Metropolitan Health Service Human Research Ethics Committee (RGS0000003673).

## 3 Results

Across 4 years, 1141 patients were enrolled: 724 (63.4%) were male with a median age of 33 years (25–41 years; Table 1). At the index presentation, most patients arrived by ambulance (853, 74.8%). 1 in 5 required intensive care (215, 18.8%). Three‐quarters of all patients were discharged home (859, 75.3%).

**TABLE 1:** Characteristics of emergency department attendances enrolled into the Emerging Drugs Network of Australia Registry, stratified by reattendance to the emergency department within 1 year of the index presentation.

Characteristics	Total cohort (*n* = 1141)	No reattendance (*n* = 1084)	Reattended (*n* = 57)	*p*
Male *n* (%)	724 (63.4)	689 (63.6)	35 (61.4)	0.74
Age median (Q1–Q3)	33 (25–41)	32 (25–41)	37 (27–42)	0.0068**
ATS *n* (%)				
1	393 (34.4)	376 (34.7)	17 (29.8)	
2	515 (45.1)	485 (44.7)	30 (52.6)	0.26
3–5	230 (20.2)	220 (20.3)	10 (17.5)	0.6731
Unknown	3 (0.3)	3 (0.3)	0 (0.0)	^a^
Arrival Mode *n* (%)				
Ambulance	853 (74.8)	802 (74.0)	51 (89.5)	—
Other arrival mode	288 (25.2)	282 (26.0)	6 (10.5)	0.0129*
Setting of exposure *n* (%)				
Primary/private residence	426 (37.3)	409 (37.7)	17 (29.8)	—
Public environment	381 (33.4)	349 (32.2)	32 (56.1)	0.0008***
Other	277 (24.3)	269 (24.8)	8 (14.0)	0.06
Unknown	57 (5.0)	57 (5.3)	0 (0.0)	^a^
ED disposition *n* (%)				
Other hospital unit	450 (39.4)	429 (39.6)	21 (36.8)	—
ICU	215 (18.8)	208 (19.2)	7 (12.3)	0.18
Home	382 (33.5)	357 (32.9)	25 (43.9)	0.07
Other disposition location^b^	94 (8.2)	90 (8.3)	4 (7.0)	^a^
Final discharge location *n* (%)				
Home	859 (75.3)	814 (75.1)	45 (78.9)	—
Other discharge location^c^	282 (24.7)	270 (24.9)	12 (21.1)	0.51

*Note:* *p < 0.05, **p < 0.01, ***p < 0.001.

Abbreviations: ATS, Australasian Triage Scale; ED, emergency department; ICU, intensive care unit.

^a^ Not included in the regression due to cell counts less than five.

^b^ Includes transfer to psychiatric facility, police or corrective services, or discharge against medical advice.

^c^ Includes transfer to other hospital or psychiatric facility, police or corrective services, or death.

NPS were detected in 93 (8.1%) index presentations, most frequently bromazolam (39, 41.9%). Illicit drugs were detected in 737 (64.6%) index presentations; most frequently methylamphetamine (620, 84.1%), gamma‐hydroxybutyrate (199, 27.0%) and 3,4‐methylenedioxymethamphetamine (63, 8.5%). Of these, 681 (92.4%) index presentations had illicit drugs without NPS detected.

**FIGURE 1:**
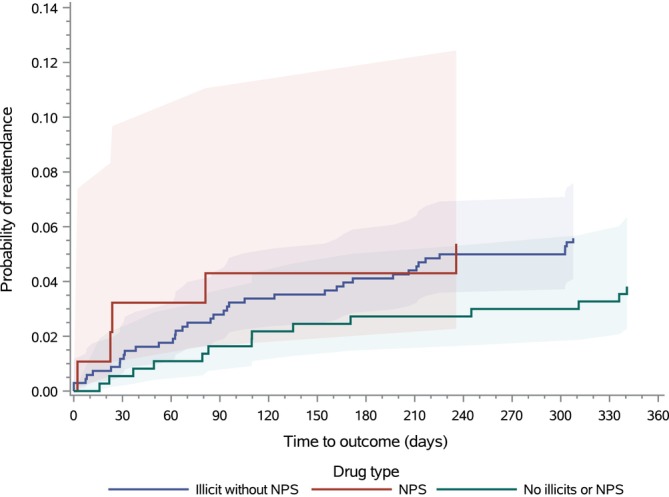
Kaplan–Meier cumulative incidence (Cumulative incidence = 1‐Kaplan Meier survival estimate) curve and 95% confidence bands of time to reattendance after index emergency department attendance for suspected severe/unusual drug exposure, stratified by confirmed type of drug detected. NPS, novel psychoactive substance. Shaded areas represent the 95% confidence band for the cumulative incidence curve of each drug type.

Overall, 57 patients (5.0%, 95% CI: 3.9%–6.4%; Figure 1) experienced 71 reattendances within 1 year. 9 patients (15.8%) reattended multiple times; 2 patients had 5 reattendances within 1 year. Reattending patients were more likely to be older, arrive by ambulance, and have a last known location or drug exposure setting within the public environment (e.g., parks, streets) (*p* < 0.05) (Table 1).

5 patients with NPS detected at index presentation reattended (5.4%, 95% CI: 2.3%–12.4%; Figure 1); 3 had the same NPS re‐detected (bromazolam), and 2 had traditional illicit drugs detected. Among the 681 patients with confirmed traditional illicit drugs only, 38 (5.6%, 95% CI: 4.1%–7.6%) reattended within 1 year. Of these, 32 (84.2%) had illicit drugs re‐detected, and 1 had an NPS detected (methcathinone). Of the patients with no confirmed NPS/traditional illicit drugs detected at index presentation (367, 32.2%), 14 reattended (3.8%, 95% CI: 2.3%–6.4%), and 3 reattendances had NPS detected (bromazolam, etizolam, 8‐aminoclonazolam). Reattendance rates did not differ between type of drug exposure.

## 4 Discussion

1 in 20 patients reattended an EDNA‐participating WA ED within 1 year for suspected severe/unusual drug exposure. No differences between one‐year reattendance rates of NPS versus illicit drug exposure were identified.

3 of 5 reattending patients with confirmed NPS exposure had bromazolam confirmed in both attendances. Exposure to NPSs are frequently unknown by the patient due to substitution or adulteration of traditional illicit drugs or counterfeit pharmaceutical products [4]. Comprehensive toxicology testing in the ED may improve awareness of NPS exposure, allow for the provision of more accurate patient follow‐up, and prevent repeated patterns of acute harm. The ED may represent an under‐utilised opportunity for harm reduction information exchange, particularly around NPS exposure, via targeted patient follow‐up and referral to alcohol and other drug services [5].

This study has limitations. EDNA employs purposive blood sampling, relies on clinical interpretation, and represents a small subset of drug‐related attendances from EDNA‐participating EDs. Therefore, results are conservative, underestimate drug‐related reattendance rates, and cannot be extrapolated beyond severe/unusual drug‐related presentations. However, a recognisable pattern of reattendance was identified. These trends and associated acute harms should be further explored through confirmed drug exposure cohorts.

## 5 Conclusion

We provide conservative but novel information on potentially preventable drug‐related ED reattendances. Access to comprehensive toxicology results provides analytic‐based opportunities to support harm reduction information exchange and reduce recurrent harm.

## Funding

EDNA is funded by a five‐year National Health and Medical Research Council (NHMRC) Ideas Grant (ROR https://ror.org/011kf5r7;0 [GNT2001107]), and additional state‐specific funding from the Mental Health Commission and the Western Australian Department of Health.

## Conflicts of Interest

The authors declare no conflicts of interest.

## Data Availability

The data that support the findings of this study are available on request from the corresponding author. The data are not publicly available due to privacy or ethical restrictions.
